# Kuru fieldwork in 1981 … and beyond

**DOI:** 10.1098/rstb.2008.4016

**Published:** 2008-11-27

**Authors:** Robert Klitzman

**Affiliations:** Columbia University722 West 168th Street, New York, NY 10032, USA

In 1981, I was fortunate to be able to conduct epidemiological fieldwork on kuru and the experience forever changed me. At the time, the prevalence and incidence had both declined markedly. Yet, clusters of cases still occurred in various villages and questions arose of whether these were the results of the last feast held in each of these areas. I trekked throughout the kuru region, examining current cases and collecting genealogies on 65 recent patients. As described more fully in a paper in *Neuroepidemiology* (Klitzman *et al.* 1984) and in a book about my fieldwork in Papua New Guinea, *The trembling mountain: a personal account of kuru, cannibals, andmad cow disease* (Klitzman 1998), I identified and described three clusters of patients, with patients in each developing kuru virtually simultaneously after having been infected at the same one or two feasts that occurred close together in time. The three pairs had incubation periods of 21, 24 and 28 years, and members of each pair did not vary by more than a year. This research suggested that the disease could therefore follow a uniform course of incubation in two or more people, even when the incubation period is over two decades. It was thus possible to determine when exposure occurred, and hence calculate precisely natural incubation periods for prions in humans—which had not been done before.

Yet I found, too, that some participants at each of these feasts had much shorter incubation periods. Hence, age and viral strains did not determine incubation period. Perhaps the initial dose of the agent or the genetics of the infected individual did. We then drew blood from the elderly survivors of multiple feasts, who, in some cases, had had their daughters and other family members succumb to the disease.

I also found that at each of these feasts almost 50 people were present. Thus, people had attended many feasts, and in their lifetimes had many opportunities to become infected.

I learned, too, about not only the epidemiology but also the anthropology of kuru—about beliefs that sorcery caused and could cure the illness. A counter-sorcerer, for instance, told me he had cured dozens of cases. His treatment consisted of first uttering an incantation, and then dispensing herbal medicines, and prescribing several behavioural changes: for one week, patients were not allowed to drink water, eat salt or touch members of the opposite sex.

But, as the disease had wiped out most of the population, people who developed headaches, backaches or a host of other minor pains now feared they had kuru. These cases, which I labelled as misdiagnoses and as hypochondriacal, presumably constituted the ‘cures’, and lent credence to the Fore belief that magic could control the disease.

Yet, none of the patients whom I had diagnosed as having kuru had responded to this therapy. I wondered how it was that he did not then perceive a problem, or feel troubled by the apparent ineffectiveness of his intervention. I asked him why some of his patients still died.

‘Very simple’, he explained, ‘they didn't follow my advice. They drank water, ate salt or touched a member of the opposite sex’.

In short, they were non-compliant. He blamed these failures not on the treatment, but on the patients. No one questioned the efficacy of the treatment itself. Though some patients worsened, the perception that others improved demonstrated that his approach could work.

‘But kuru is decreasing’, I pointed out to him, ‘because cannibalism is no longer practised’.

‘No’, I was told, ‘fewer die of the disease because the sorcerers have finally heard our pleas, and seen the evil they have done. Besides, only the older generation knows the poison. The younger generation does not.’

‘How come anyone still dies then?’ I asked, thinking I would stump him.

‘Because a few old timers are still left who still practice the sorcery’.

‘But small children no longer die of the disease’, I said, summarizing the epidemiological data. ‘Each year, the youngest people to die are older, since the last feasts are further in the past. The youngest patients used to be children, and now are adults.’

‘That's because children haven't lived long enough to anger the sorcerers. Others still die. My own brother died of kuru last year. If you white men think you know what causes kuru, why haven't you cured it? We've cured it. You haven't!’

The Fore all believed that counter-sorcery worked, and that these causal theories were correct. The group chose the theory that it thought yielded a cure, and thus worked.

I felt frustrated, but the coherence and strength of the Fore counter-sorcerer's logic impressed me.

Years later, training as a doctor, I again heard similar arguments to explain the successes or failures of treatments. In American medicine and psychiatry, doctors often blamed treatment failures on patients' poor compliance or low motivation, rather than questioning the efficacy or limitations of the therapies employed. When patients failed to improve, we rarely questioned the potential efficacy of the intervention itself. We rarely acknowledged the potential roles of beliefs and magic—as in the placebo effect—in what we did.

Since I returned from Papua New Guinea, my experiences with kuru have continued to inspire me. The insights I gained there about not only kuru have profoundly shaped my research and writing. I explored the roles of beliefs in medical and psychiatric training—how competing views about causes of diseases shape what doctors do (Klitzman 1989,1995).

The year when I returned from Papua New Guinea and started medical school, the first cases of HIV/AIDS appeared; and my experiences with the complexities of how a culture responds to an epidemic in its midst—the fears, the desperate searches for cures, and the beliefs about these—again impelled my work ([Bibr bib3Q9][Bibr bib6Q9]).

My recent book, *When doctors become patients* (Klitzman 2008), examines further how doctors, when they get sick themselves, shift how they see the power, magic and beliefs they possessed—now viewing these from the other side.

In all, over the past quarter century, what the Fore and their neighbours taught me through their openness has shaped all I have done, and I am sure will continue to do so for the rest of my life.
